# Author Correction: Adipose tissue retains an epigenetic memory of obesity after weight loss

**DOI:** 10.1038/s41586-025-08909-z

**Published:** 2025-07-07

**Authors:** Laura C. Hinte, Daniel Castellano-Castillo, Adhideb Ghosh, Kate Melrose, Emanuel Gasser, Falko Noé, Lucas Massier, Hua Dong, Wenfei Sun, Anne Hoffmann, Christian Wolfrum, Mikael Rydén, Niklas Mejhert, Matthias Blüher, Ferdinand von Meyenn

**Affiliations:** 1 Laboratory of Nutrition and Metabolic Epigenetics, Institute of Food, Nutrition and Health, Department of Health Sciences and Technology, ETH Zurich, Zurich, Switzerland; 2 Biomedicine Programme, Life Science Zurich Graduate School, Zurich, Switzerland; 3 Functional Genomics Center Zurich, ETH Zurich and University Zurich, Zurich, Switzerland; 4 Laboratory of Translational Nutrition Biology, Institute of Food, Nutrition and Health, Department of Health Sciences and Technology, ETH Zurich, Zurich, Switzerland; 5 Department of Medicine Huddinge, Karolinska Institutet, Karolinska University Hospital Huddinge, Stockholm, Sweden; 6 Helmholtz Institute for Metabolic, Obesity and Vascular Research (HI-MAG), Helmholtz Zentrum München, University of Leipzig and University Hospital Leipzig, Leipzig, Germany; 7 Medical Department III – Endocrinology, Nephrology, Rheumatology, University of Leipzig Medical Center, Leipzig, Germany; 8 Present Address: Medical Oncology Department, Virgen de la Victoria University Hospital, Málaga Biomedical Research Institute (IBIMA)-CIMES-UMA, Málaga, Spain; 9 Present Address: Helmholtz Institute for Metabolic, Obesity and Vascular Research (HI-MAG), Helmholtz Zentrum München, University of Leipzig and University Hospital Leipzig, Leipzig, Germany; 10 Present Address: Stem Cell Bio Regenerative Med Institute, Stanford University, Stanford, CA USA; 11 Present Address: Department of Bioengineering, Stanford University, Stanford, CA USA

**Keywords:** Endocrine system and metabolic diseases, Physiology, Epigenetics

Correction to: *Nature* 10.1038/s41586-024-08165-7 Published online 18 November 2024

In the version of the article initially published, in Fig. 2e and f the label “HCC” should have read “HHC” and has now been corrected. In Extended Data Fig. 5z, the label “CC” should have read “CC_s”. In Extended Data Fig. 10j the “HCH” label was illegible and has been amended. Additionally, the Source data for this figure has been corrected because one column reflected the data for HHC mice (displayed in Extended Data Fig. 5m) and not HCH mice. In Extended Data Fig. 10k the label “HHC” should have read “HCH”. In the “Epigenetic obesogenic memory in mice” section, in the sentence now reading “Many of these epigenetic changes were also reflected in the translatome (Fig. 3h) and nuclear transcriptome (Fig. 2g,h)”, the citation to Fig. 2g,h originally referred to Fig. 2f. In the “Metabolic memory primes adipocytes” section, a citation to Fig. 2f has been removed from the sentence, which now reads “Interestingly, these overlapped with promoters and enhancers carrying epigenetic memory (Figs. 3h and 5k,l)”. These errors, which do not affect the underlying data and interpretations of the article, have been corrected in the HTML and PDF versions of the article.

