# Supplementary material for: Adipose tissue retains an epigenetic memory of obesity after weight loss
Source: Nature. 2024 Nov 18;636(8042):457–65. doi: 10.1038/s41586-024-08165-7 (PMC11634781; doi:10.1038/s41586-024-08165-7)
Supplement: Supplementary file 1 — Reporting Summary [file 41586_2024_8165_MOESM1_ESM.pdf]

nature portfolio

Corresponding author(s): Ferdinand von Meyenn

Last updated by author(s): Sep 11, 2024

Reporting Summary

Nature Portfolio wishes to improve the reproducibility of the work that we publish. This form provides structure for consistency and transparency in reporting. For further information on Nature Portfolio policies, see our Editorial Policies and the Editorial Policy Checklist.

Statistics

For all statistical analyses, confirm that the following items are present in the figure legend, table legend, main text, or Methods section.

n/a	Confirmed	
☐	☒	The exact sample size (*n*) for each experimental group/condition, given as a discrete number and unit of measurement
☐	☒	A statement on whether measurements were taken from distinct samples or whether the same sample was measured repeatedly
☐	☒	The statistical test(s) used AND whether they are one- or two-sided<br>*Only common tests should be described solely by name; describe more complex techniques in the Methods section.*
☒	☐	A description of all covariates tested
☐	☒	A description of any assumptions or corrections, such as tests of normality and adjustment for multiple comparisons
☐	☒	A full description of the statistical parameters including central tendency (e.g. means) or other basic estimates (e.g. regression coefficient) AND variation (e.g. standard deviation) or associated estimates of uncertainty (e.g. confidence intervals)
☐	☒	For null hypothesis testing, the test statistic (e.g. *F*, *t*, *r*) with confidence intervals, effect sizes, degrees of freedom and *P* value noted<br>*Give P values as exact values whenever suitable.*
☒	☐	For Bayesian analysis, information on the choice of priors and Markov chain Monte Carlo settings
☒	☐	For hierarchical and complex designs, identification of the appropriate level for tests and full reporting of outcomes
☒	☐	Estimates of effect sizes (e.g. Cohen's *d*, Pearson's *r*), indicating how they were calculated

Our web collection on statistics for biologists contains articles on many of the points above.

Software and code

Policy information about availability of computer code

Data collection	Sable Systems Promethion ExpeData 1.9.27b Software; BioTek Gen5 Software; 3DHISTECH Slide Viewer 2; Adiposoft as plugin in Fiji Image J; a NovaSeq 6000 and a NovaSeqX were used for sequencing
Data analysis	Analysis of non-sequening data: GraphPad Prism v9.5.1<br>Analysis of sequencing data:<br><br>Single nucleus RNA seq for mouse:<br>The 10x Genomics Cell Ranger v.6.1.2 pipeline was used for demultiplexing, read alignment to reference genome The R packages Seurat v4.1.0; scDblFinder and Scpubr were used for analysis.<br><br>Single nucleus RNA seq (human):<br>The 10x Genomics Cell Ranger v.7.2.0 pipeline was used for demultiplexing, read alignment to reference genome The R packages Seurat v4.1.0; scDblFinder and Scpubr were used for analysis. SNP-calling and demultiplexing was performed using cellsnp-lite and vireo.<br><br>mouse TRAP-seq:<br>Quality control of the raw reads was performed using FastQC v0.11.9. Raw reads were trimmed using TrimGalore v0.6.6 (https://github.com/FelixKrueger/TrimGalore). Filtered reads were aligned against the reference mouse genome assembly mm10 using HISAT2 v2.2.1. Raw gene counts were quantified using the featureCounts program of subread v2.0.1. Differential expression analysis was performed using the R package EdgeR.<br><br>CUT&Tag and ATAC-seq:

Quality control of the raw sequencing reads was performed using FastQC v0.11.9. Raw reads were trimmed off low-quality bases and adapter sequences using TrimGalore v0.6.6 (https://github.com/FelixKrueger/TrimGalore). Aligned bam files were sorted based on chromosomal coordinates using the sort function of samtools v1.13. Sorted bam files were summarized into bedgraph files using the genomecov function of bedtools v2.30. Peaks were called from CUT&Tag-seq and ATAC-seq libraries on individual bedgraph files using SEACR v1.3. Called peaks were combined to generate a union peak list and quantified using the R package chromVAR v1.16 generating a raw peak count matrix. MOFA2 v1.4.0 was used to identify variation and ChromHMM v1.22 for identifiying chromatin states.
Peaks were annotated with ChIPSeeker and differential analysis was performed using EdgeR.

Gene set enrichment:
The package enrichR was used.

For manuscripts utilizing custom algorithms or software that are central to the research but not yet described in published literature, software must be made available to editors and reviewers. We strongly encourage code deposition in a community repository (e.g. GitHub). See the Nature Portfolio guidelines for submitting code & software for further information.

Data

Policy information about availability of data

All manuscripts must include a data availability statement. This statement should provide the following information, where applicable:

- Accession codes, unique identifiers, or web links for publicly available datasets
- A description of any restrictions on data availability
- For clinical datasets or third party data, please ensure that the statement adheres to our policy

All mouse sequencing data that support the findings of this study have been deposited on GEO, with the accession code GSE236580 (token for reviewers: qtcrauuovrghlcr). Human snRNAseq data from MTSS and LTSS studies are available upon request from CW and MB. Human snRNAseq data from the NEFA study is available upon request from FvM, MR and NM.

Research involving human participants, their data, or biological material

Policy information about studies with human participants or human data. See also policy information about sex, gender (identity/presentation), and sexual orientation and race, ethnicity and racism.

Reporting on sex and gender	The human studies MTSS (3 male, 10 female) and LTSS (4 male, 6 female) are part of the Leipzig Obesity Biobank (LOBB) maintained by Dr. Matthias Blüher. The human study NEFA (15 female) was run by Mikalel Rydén and Niklas Mejhert at the Karolinska Institute.
Reporting on race, ethnicity, or other socially relevant groupings	N/A
Population characteristics	MTSS: Only individuals that lost 25% of BMI (31.26 ± 4.77 % reduction) and lean, healthy (BMI < 27) controls were selected from the MTSS study for this study.<br>LTSS study: Only individuals that lost 25% of BMI (26.21 ± 0.92 % reduction) and lean, healthy (BMI < 27) controls were selected from the LTSS study for this study.<br>NEFA study: Only individuals that lost 25% of BMI (34.22 ± 7.61 % reduction) and lean, healthy (BMI < 27) controls were selected from the NEFA study for this study.
Recruitment	MTSS and LTSS studies are part of the Leipzig Obesity Biobank (LOBB). The NEFA study is registered with clinical trial number: NCT01727245
Ethics oversight	The human studies MTSS and LTSS was conducted in accordance with the Declaration of Helsinki and approved by the Ethics Committee of the University of Leipzig (approval number: 159-12-21052012). Written informed consent was obtained from all subjects involved in the study prior to surgery.<br>The human study NEFA was conducted in accordance with the with the Declaration of Helsinki and approved by the Ethics Committee of the Karolinska Institute, Stockholm (approval number: 2011/1002-31/1).

Note that full information on the approval of the study protocol must also be provided in the manuscript.

Field-specific reporting

Please select the one below that is the best fit for your research. If you are not sure, read the appropriate sections before making your selection.

☒ Life sciences ☐ Behavioural & social sciences ☐ Ecological, evolutionary & environmental sciences

For a reference copy of the document with all sections, see nature.com/documents/nr-reporting-summary-flat.pdf

Life sciences study design

All studies must disclose on these points even when the disclosure is negative.

Sample size	No sample size calculations were performed. Samples sizes were chosen to allow sufficient statistical analysis to be performed. For the human cohort sample size was dependent on the availability of samples of subjects meeting the selection criteria (e.g. weight loss, healthy).
Data exclusions	snRNAseq data from two donors were exlcuded after consultation with surgeons because they either contained more thant 50% B-cells or no adipocytes. This is explained in the Methods section.
Replication	Experimental findings were verified by biological replicates. Each experiment was performed multiple times. Replication attempts were successful and well correlated. At least two independent biological replicates were included in the RNA-seq, ATAC-seq and CUT&Tag (like ChIPseq) experiments. For snRNAseq 5 of mice individual biological replicates were pooled per condition. For human snRNAseq samples belonging to one group and time point were pooled and later SNP demultiplexed.
Randomization	Samples were not randomised and were allocated into experimental groups by condition.
Blinding	The investigators were not blinded to the mice as they themselves were treating and sacrificing the mice. However, investigators were blinded during mouse sample processing. Investigators were not blinded to the sample identity for sequencing data (human and mouse) as sequencing data was produced by objective quantitative methods. For histological image quantification the investigators analysing adipocytes and imaging sections were blinded.

Reporting for specific materials, systems and methods

We require information from authors about some types of materials, experimental systems and methods used in many studies. Here, indicate whether each material, system or method listed is relevant to your study. If you are not sure if a list item applies to your research, read the appropriate section before selecting a response.

Materials & experimental systems

n/a	Involved in the study	
☐	☒	Antibodies
☒	☐	Eukaryotic cell lines
☒	☐	Palaeontology and archaeology
☐	☒	Animals and other organisms
☒	☐	Clinical data
☒	☐	Dual use research of concern
☒	☐	Plants

Methods

n/a	Involved in the study	
☐	☒	ChIP-seq
☒	☐	Flow cytometry
☒	☐	MRI-based neuroimaging

Antibodies

Antibodies used	anti-H3K4me3 (abcam, #ab8580), anti-H3K27me3 (Cell Signaling Technology, #C36B11), anti-H3K27ac (abcam, #ab4729), anti-H3K4me1 (abcam, #ab8895)
Validation	anti-H3K4me3 (abcam, #ab8580): has been validated by abcam for human and cow and is predicted for mouse for ChIP; has been validated in various publications for mouse and other species for example in: https://doi.org/10.1016/j.molcel.2022.03.009;<br>anti-H3K27me3 (Cell Signaling Technology, #C36B11): has been validate for human, mouse, rat and monkey by Cell Signaling Technology for ChIP;<br>anti-H3K27ac (abcam, #ab4729): has been validate for human, mouse, rat, and cow by abcam for ChIP<br>anti-H3K4me1 (abcam, #ab8895): has been validate for human, mouse, rat, and cow by abcam for ChIP

Animals and other research organisms

Policy information about studies involving animals; ARRIVE guidelines recommended for reporting animal research, and Sex and Gender in Research

Laboratory animals	C57NL/6-Tg(Adipoq CreER)426Biat/N x B6;129S6-Gt(ROSA)26Sortm2(CAG-NuTRAP)Evdr/J were used. C57BL/6J DIO (#380050) and DIO control (#380056) male mice were obtained from Jackson Laboratory (USA).<br>All mice were kept on a 12-h/12-h light/dark cycle and 20-60% (23℃) humidity in individually ventilated cages in groups of between two and five mice in a pathogen-free animal facility of SLA ETH Zurich.
Wild animals	No wild animals were used in this study.
Reporting on sex	Only male mice were used in this study.

Field-collected samples	No field collected samples were used in this study.
Ethics oversight	All animal experiments were approved by the cantonal veterinary office Zurich.

Note that full information on the approval of the study protocol must also be provided in the manuscript.

Plants

Seed stocks	*Report on the source of all seed stocks or other plant material used. If applicable, state the seed stock centre and catalogue number. If plant specimens were collected from the field, describe the collection location, date and sampling procedures.*
Novel plant genotypes	*Describe the methods by which all novel plant genotypes were produced. This includes those generated by transgenic approaches, gene editing, chemical/radiation-based mutagenesis and hybridization. For transgenic lines, describe the transformation method, the number of independent lines analyzed and the generation upon which experiments were performed. For gene-edited lines, describe the editor used, the endogenous sequence targeted for editing, the targeting guide RNA sequence (if applicable) and how the editor was applied.*
Authentication	*Describe any authentication procedures for each seed stock used or novel genotype generated. Describe any experiments used to assess the effect of a mutation and, where applicable, how potential secondary effects (e.g. second site T-DNA insertions, mosiacism, off-target gene editing) were examined.*

ChIP-seq

Data deposition

☒ Confirm that both raw and final processed data have been deposited in a public database such as GEO.

☒ Confirm that you have deposited or provided access to graph files (e.g. BED files) for the called peaks.

Data access links
May remain private before publication.

All mouse sequencing data that support the findings of this study have been deposited on GEO, with the accession code GSE236580 (token for reviewers: qtcrauuovrghlcr).

Files in database submission

Mouse data: BED and fastq (in tar format) files for all indvidual CUT&Tag and ATAC-seq;

ATAC-seq:
GSM7558081 C_short_ATAC_1
GSM7558082 H_short_ATAC_1
GSM7558083 CC_short_ATAC_1
GSM7558085 HC_short_ATAC_1
GSM7558086 C_short_ATAC_2
GSM7558087 H_short_ATAC_2
GSM7558088 CC_short_ATAC_2
GSM7558090 HC_short_ATAC_2
GSM7558091 C_short_ATAC_3
GSM7558092 H_short_ATAC_3
GSM7558093 CC_short_ATAC_3
GSM7558094 HC_short_ATAC_3
GSM7558096 CC_long_ATAC_1
GSM7558097 HH_long_ATAC_1
GSM7558098 CCC_long_ATAC_1
GSM7558099 HHC_long_ATAC_1
GSM7558101 CC_long_ATAC_2
GSM7558102 HH_long_ATAC_2
GSM7558103 CCC_long_ATAC_2
GSM7558104 HHC_long_ATAC_2
GSM7558105 HH_long_ATAC_3
GSM7558107 HHC_long_ATAC_3

CUT&Tag:
GSM7558140 CC_long_H3K4me1_1_AdipoERCre
GSM7558142 HH_long_H3K4me1_1_AdipoERCre
GSM7558143 CCC_long_H3K4me1_1_AdipoERCre
GSM7558144 HHC_long_H3K4me1_1_AdipoERCre
GSM7558146 CC_long_H3K4me1_2_AdipoERCre
GSM7558147 HH_long_H3K4me1_2_AdipoERCre
GSM7558148 HHC_long_H3K4me1_2_AdipoERCre
GSM7558150 HH_long_H3K4me1_3_AdipoERCre
GSM7558151 CCC_long_H3K4me1_3_AdipoERCre
GSM7558152 HHC_long_H3K4me1_3_AdipoERCre
GSM7558153 CC_long_H3K4me3_1_AdipoERCre
GSM7558155 CCC_long_H3K4me3_1_AdipoERCre
GSM7558156 HHC_long_H3K4me3_1_AdipoERCre
GSM7558157 CC_long_H3K4me3_2_AdipoERCre

GSM7558158 HH_long_H3K4me3_2_AdipoERCre
GSM7558159 HHC_long_H3K4me3_2_AdipoERCre
GSM7558161 HH_long_H3K4me3_3_AdipoERCre
GSM7558162 CCC_long_H3K4me3_3_AdipoERCre
GSM7558163 HHC_long_H3K4me3_3_AdipoERCre
GSM7558164 CC_long_H3K27ac_1_AdipoERCre
GSM7558165 HH_long_H3K27ac_1_AdipoERCre
GSM7558167 CCC_long_H3K27ac_1_AdipoERCre
GSM7558168 HHC_long_H3K27ac_1_AdipoERCre
GSM7558169 CC_long_H3K27ac_2_AdipoERCre
GSM7558170 HH_long_H3K27ac_2_AdipoERCre
GSM7558172 HHC_long_H3K27ac_2_AdipoERCre
GSM7558173 HH_long_H3K27ac_3_AdipoERCre
GSM7558174 CCC_long_H3K27ac_3_AdipoERCre
GSM7558175 HHC_long_H3K27ac_3_AdipoERCre
GSM7558177 CC_long_H3K27me3_1_AdipoERCre
GSM7558178 CCC_long_H3K27me3_1_AdipoERCre
GSM7558179 HHC_long_H3K27me3_1_AdipoERCre
GSM7558181 CC_long_H3K27me3_2_AdipoERCre
GSM7558182 HH_long_H3K27me3_2_AdipoERCre
GSM7558183 HHC_long_H3K27me3_2_AdipoERCre
GSM7558184 HH_long_H3K27me3_3_AdipoERCre
GSM7558186 CCC_long_H3K27me3_3_AdipoERCre
GSM7558187 HHC_long_H3K27me3_3_AdipoERCre
GSM7558188 C_short_H3K4me1_1_AdipoERCre
GSM7558189 H_short_H3K4me1_1_AdipoERCre
GSM7558190 CC_short_H3K4me1_1_AdipoERCre
GSM7558192 HC_short_H3K4me1_1_AdipoERCre
GSM7558193 C_short_H3K4me1_2_AdipoERCre
GSM7558194 H_short_H3K4me1_2_AdipoERCre
GSM7558195 CC_short_H3K4me1_2_AdipoERCre
GSM7558196 HC_short_H3K4me1_2_AdipoERCre
GSM7558198 C_short_H3K4me1_3_AdipoERCre
GSM7558199 H_short_H3K4me1_3_AdipoERCre
GSM7558200 CC_short_H3K4me1_3_AdipoERCre
GSM7558202 HC_short_H3K4me1_3_AdipoERCre
GSM7558203 C_short_H3K4me3_1_AdipoERCre
GSM7558204 H_short_H3K4me3_1_AdipoERCre
GSM7558206 CC_short_H3K4me3_1_AdipoERCre
GSM7558207 HC_short_H3K4me3_1_AdipoERCre
GSM7558208 C_short_H3K4me3_2_AdipoERCre
GSM7558209 H_short_H3K4me3_2_AdipoERCre
GSM7558211 CC_short_H3K4me3_2_AdipoERCre
GSM7558212 HC_short_H3K4me3_2_AdipoERCre
GSM7558213 C_short_H3K4me3_3_AdipoERCre
GSM7558214 H_short_H3K4me3_3_AdipoERCre
GSM7558215 CC_short_H3K4me3_3_AdipoERCre
GSM7558217 HC_short_H3K4me3_3_AdipoERCre
GSM7558218 C_short_H3K27ac_1_AdipoERCre
GSM7558219 H_short_H3K27ac_1_AdipoERCre
GSM7558220 CC_short_H3K27ac_1_AdipoERCre
GSM7558222 HC_short_H3K27ac_1_AdipoERCre
GSM7558223 C_short_H3K27ac_2_AdipoERCre
GSM7558224 H_short_H3K27ac_2_AdipoERCre
GSM7558226 CC_short_H3K27ac_2_AdipoERCre
GSM7558227 HC_short_H3K27ac_2_AdipoERCre
GSM7558228 C_short_H3K27ac_3_AdipoERCre
GSM7558229 H_short_H3K27ac_3_AdipoERCre
GSM7558231 HC_short_H3K27ac_3_AdipoERCre
GSM7558232 C_short_H3K27me3_1_AdipoERCre
GSM7558233 H_short_H3K27me3_1_AdipoERCre
GSM7558234 CC_short_H3K27me3_1_AdipoERCre
GSM7558235 HC_short_H3K27me3_1_AdipoERCre
GSM7558236 C_short_H3K27me3_2_AdipoERCre
GSM7558238 H_short_H3K27me3_2_AdipoERCre
GSM7558239 CC_short_H3K27me3_2_AdipoERCre
GSM7558240 HC_short_H3K27me3_2_AdipoERCre
GSM7558241 C_short_H3K27me3_3_AdipoERCre
GSM7558242 CC_short_H3K27me3_3_AdipoERCre
GSM7558244 HC_short_H3K27me3_3_AdipoERCre

Genome browser session
(e.g. UCSC)

No genome browser session was created.

Methodology

Replicates	CUT and TAG : 3 biological replicates for C, CC_s, H, HC, HH, HHC for each hPTM, 2 biological replicates for CC_l and CCC for each hPTM.
Sequencing depth	Minimum of of 6 million reads per sample. Paired end sequencing, with each read 150 bp length.
Antibodies	anti-H3K4me3 (abcam, #ab8580), anti-H3K27me3 (Cell Signaling Technology, #C36B11), anti-H3K27ac (abcam, #ab4729), anti-H3K4me1 (abcam, #ab8895)
Peak calling parameters	Peaks were called from CUT&Tag-seq and ATAC-seq libraries on individual bedgraph files using SEACR v1.3 in stringent mode with a peak calling threshold of 0.01.
Data quality	Analysis code for mouse data is available on GitHub (https://github.com/vonMeyennLab/AT_memory)
Software	Analysis code for mouse data is available on GitHub (https://github.com/vonMeyennLab/AT_memory)
