# Supplementary figures and images for: Adipose tissue retains an epigenetic memory of obesity after weight loss
Source: Nature. 2024 Nov 18;636(8042):457–65. doi: 10.1038/s41586-024-08165-7 (PMC11634781; doi:10.1038/s41586-024-08165-7)

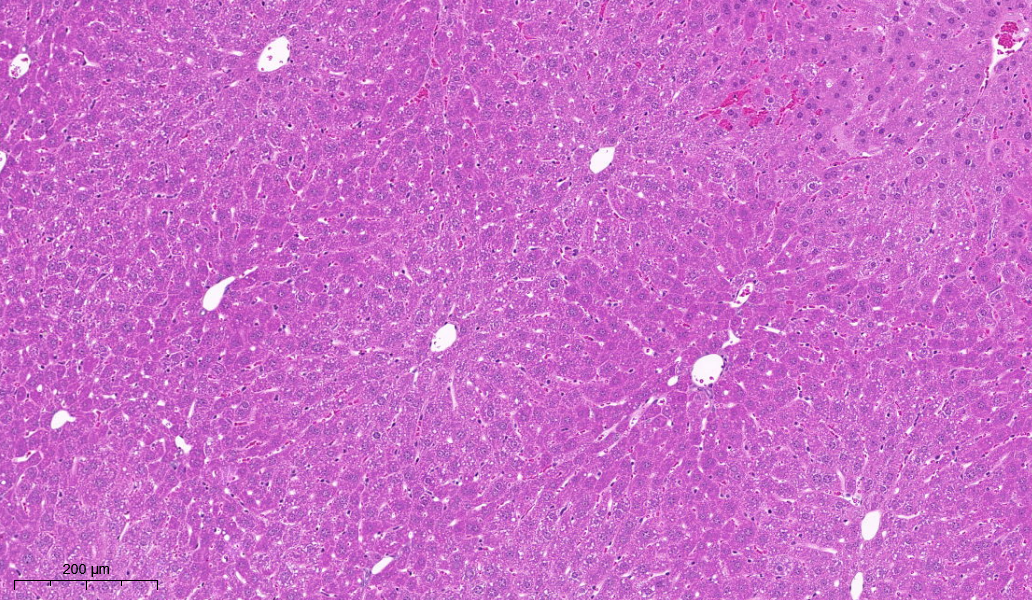

Supplement: Supplementary file 3 — Source Data Figs. 1–5 and Source Data Extended Data Figs. 1–10. [file 41586_2024_8165_MOESM3_ESM.zip › 2023-01-01106D-f1-5;sf1-10/Images/ExtendedData_Fig10/CCH.LIVER.HE_10.0x.tif]

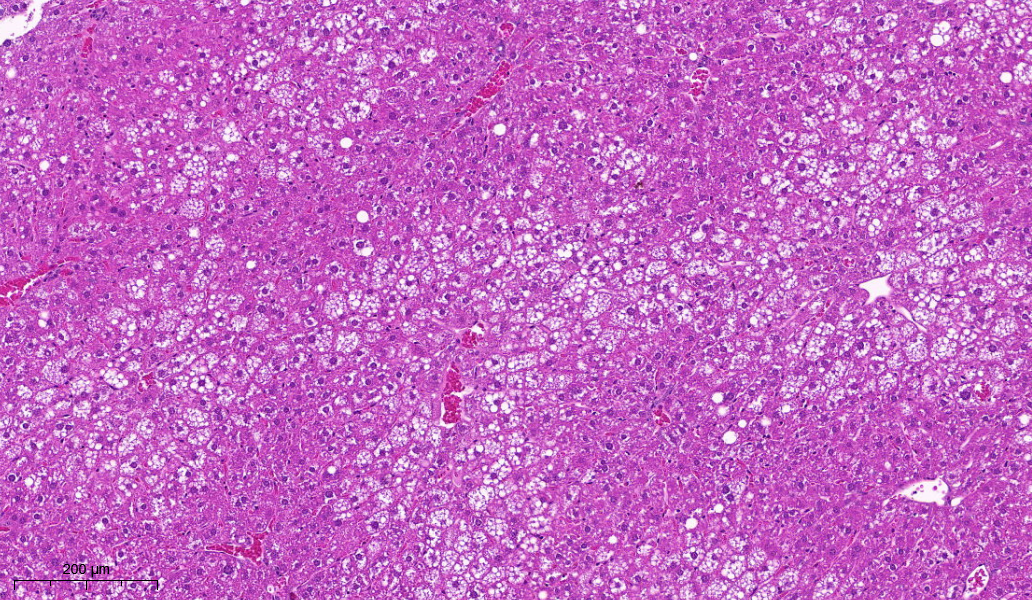

Supplement: Supplementary file 3 — Source Data Figs. 1–5 and Source Data Extended Data Figs. 1–10. [file 41586_2024_8165_MOESM3_ESM.zip › 2023-01-01106D-f1-5;sf1-10/Images/ExtendedData_Fig10/HCH.LIVER.HE_10.0x.tif]

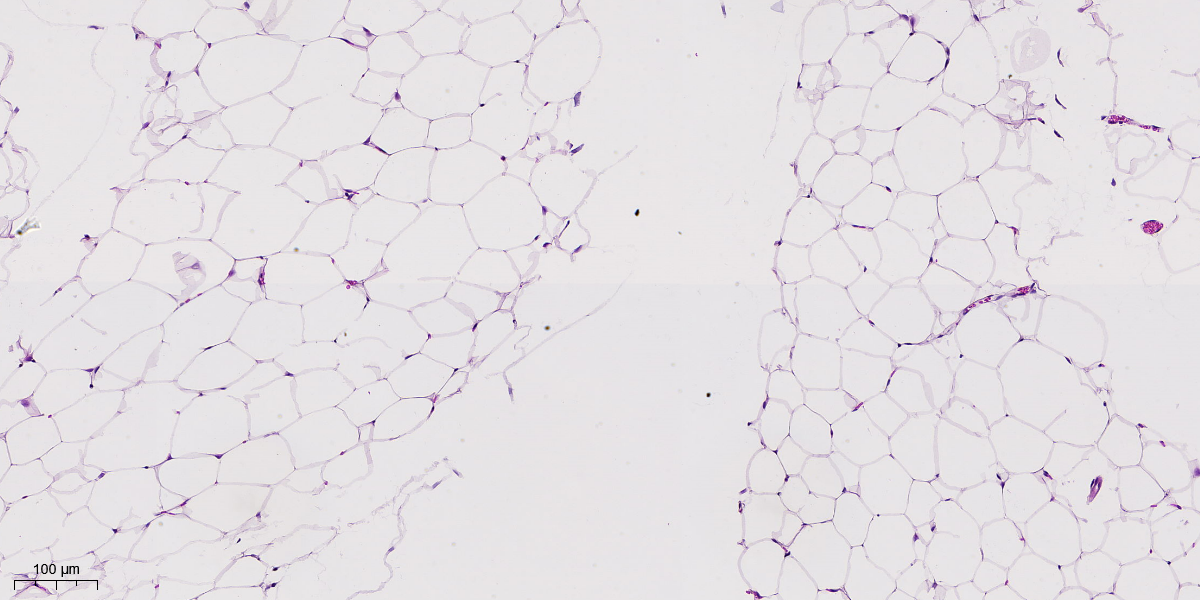

Supplement: Supplementary file 3 — Source Data Figs. 1–5 and Source Data Extended Data Figs. 1–10. [file 41586_2024_8165_MOESM3_ESM.zip › 2023-01-01106D-f1-5;sf1-10/Images/ExtendedData_Fig5/CC_l_epiAT.tif]

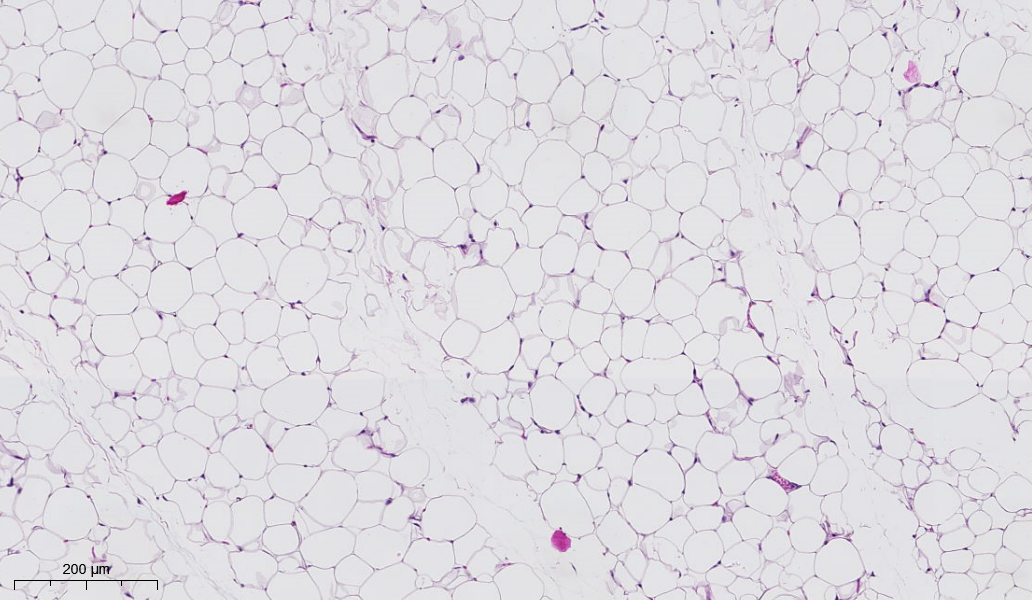

Supplement: Supplementary file 3 — Source Data Figs. 1–5 and Source Data Extended Data Figs. 1–10. [file 41586_2024_8165_MOESM3_ESM.zip › 2023-01-01106D-f1-5;sf1-10/Images/ExtendedData_Fig5/CC_l_ingAT.tif]

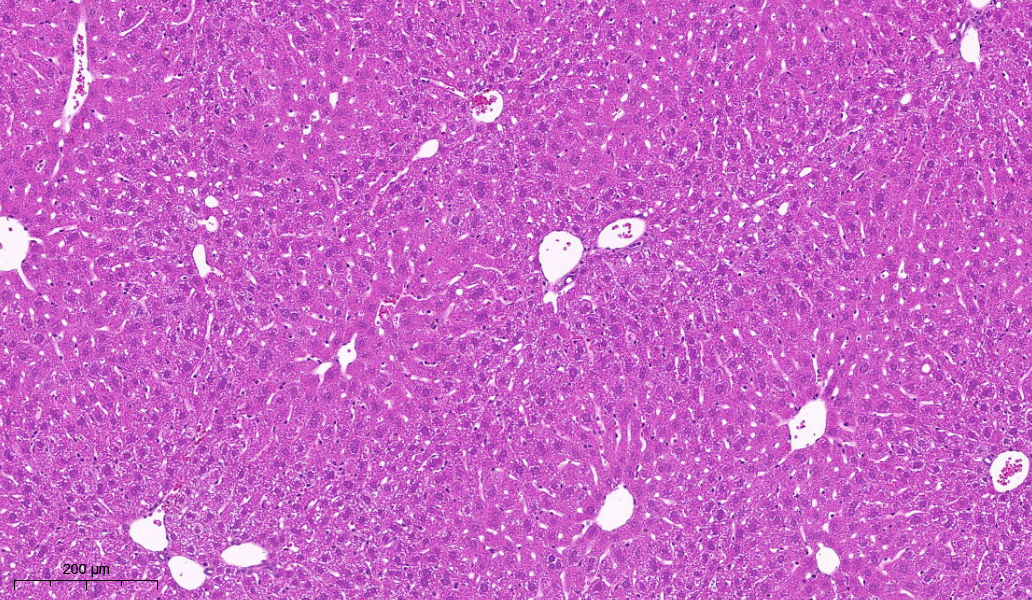

Supplement: Supplementary file 3 — Source Data Figs. 1–5 and Source Data Extended Data Figs. 1–10. [file 41586_2024_8165_MOESM3_ESM.zip › 2023-01-01106D-f1-5;sf1-10/Images/ExtendedData_Fig5/CC_l_Liver.tif]

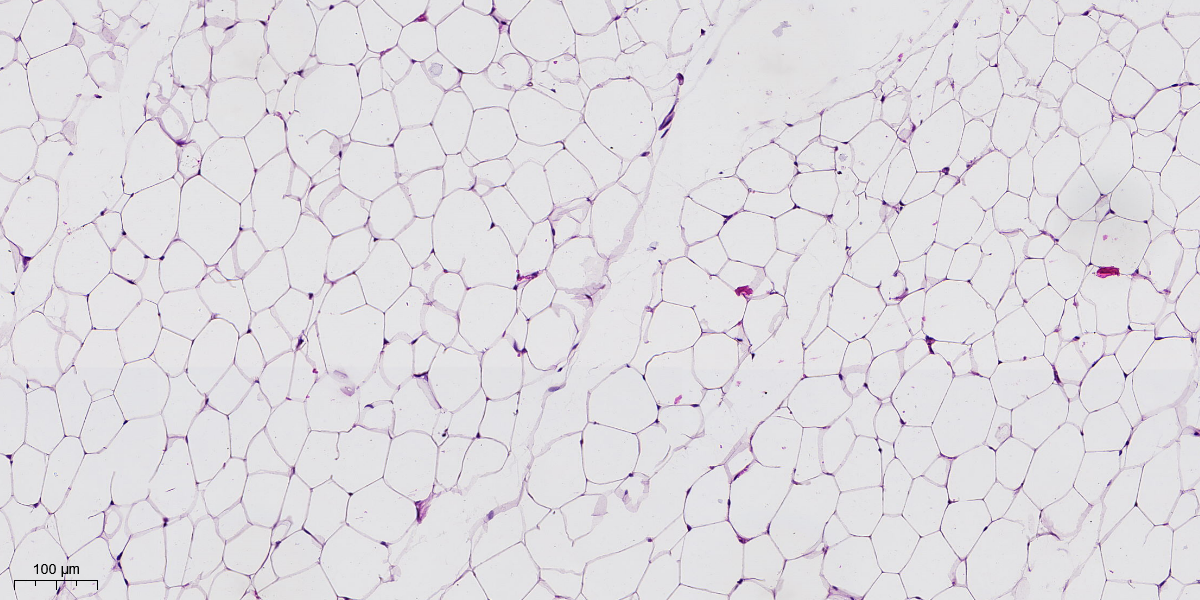

Supplement: Supplementary file 3 — Source Data Figs. 1–5 and Source Data Extended Data Figs. 1–10. [file 41586_2024_8165_MOESM3_ESM.zip › 2023-01-01106D-f1-5;sf1-10/Images/ExtendedData_Fig5/CC_s_epiAT.tif]

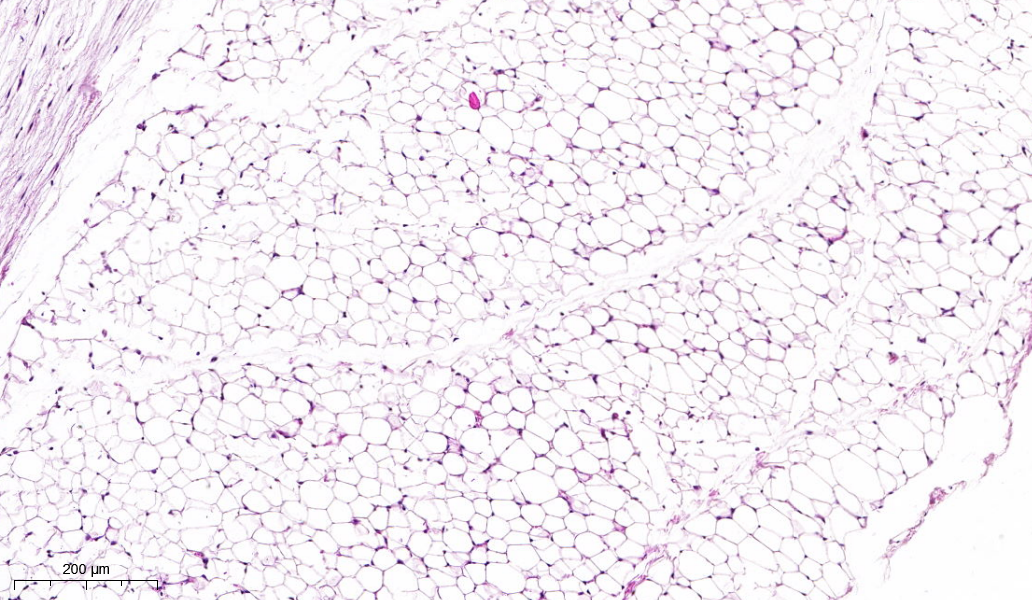

Supplement: Supplementary file 3 — Source Data Figs. 1–5 and Source Data Extended Data Figs. 1–10. [file 41586_2024_8165_MOESM3_ESM.zip › 2023-01-01106D-f1-5;sf1-10/Images/ExtendedData_Fig5/CC_s_ingAt.tif]

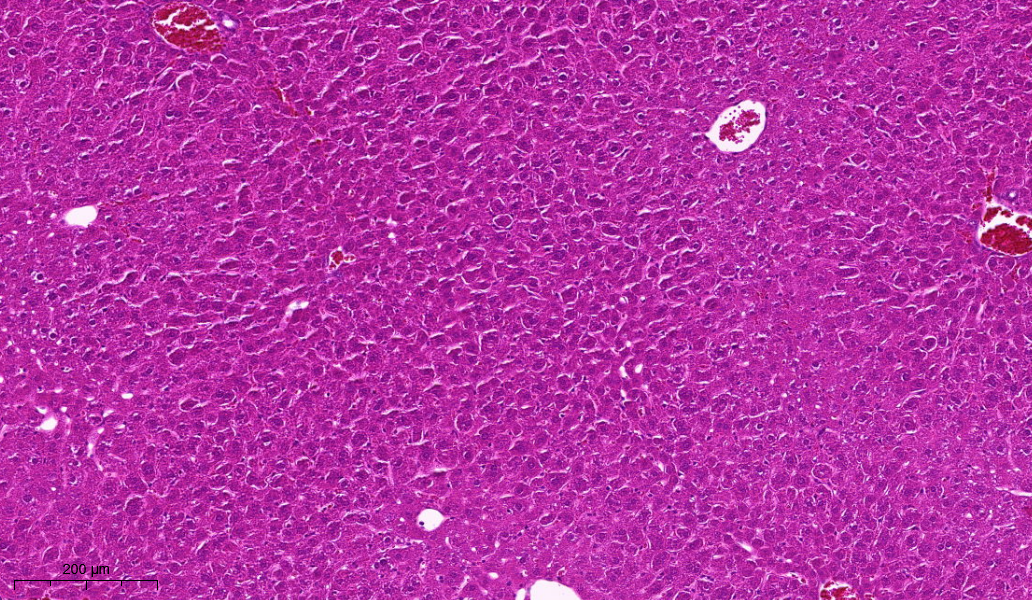

Supplement: Supplementary file 3 — Source Data Figs. 1–5 and Source Data Extended Data Figs. 1–10. [file 41586_2024_8165_MOESM3_ESM.zip › 2023-01-01106D-f1-5;sf1-10/Images/ExtendedData_Fig5/CC_s_Liver.tif]

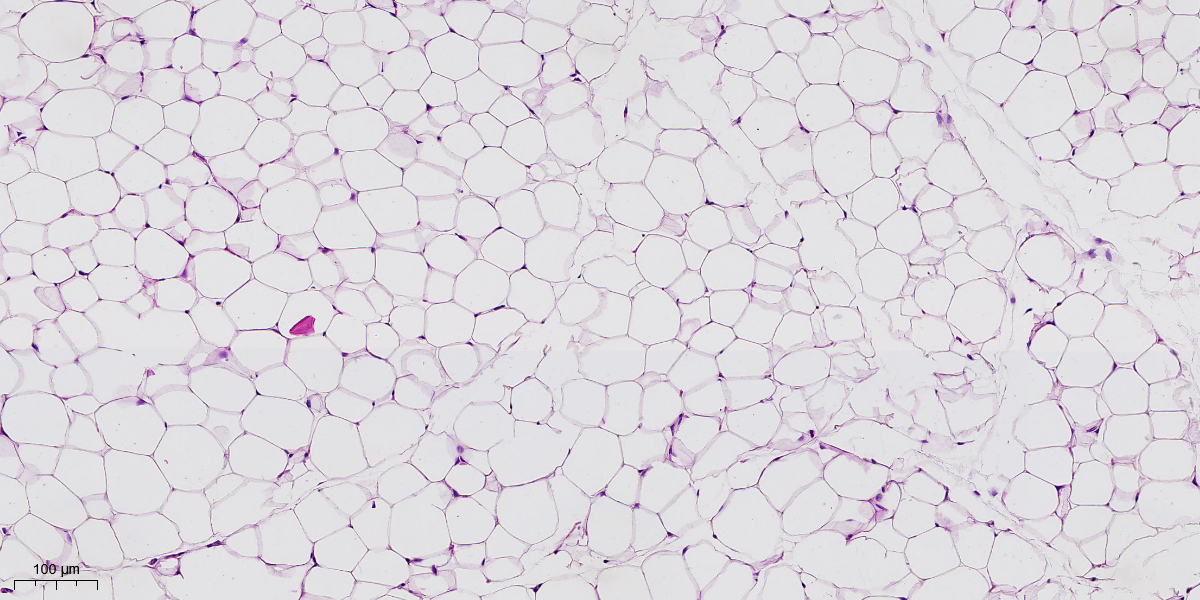

Supplement: Supplementary file 3 — Source Data Figs. 1–5 and Source Data Extended Data Figs. 1–10. [file 41586_2024_8165_MOESM3_ESM.zip › 2023-01-01106D-f1-5;sf1-10/Images/ExtendedData_Fig5/CCC_epiAT.tif]

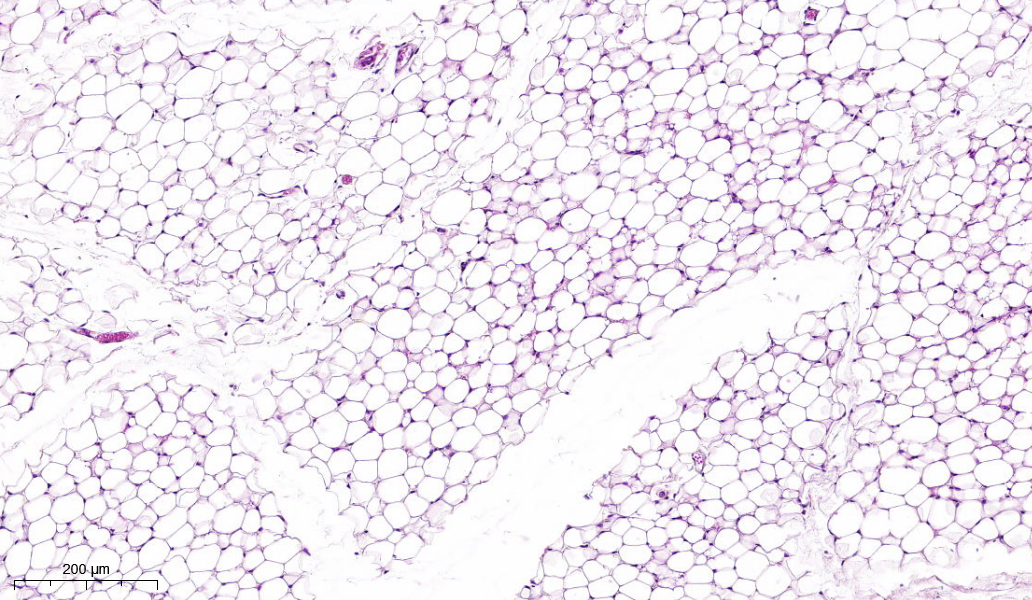

Supplement: Supplementary file 3 — Source Data Figs. 1–5 and Source Data Extended Data Figs. 1–10. [file 41586_2024_8165_MOESM3_ESM.zip › 2023-01-01106D-f1-5;sf1-10/Images/ExtendedData_Fig5/CCC_ingAt.tif]

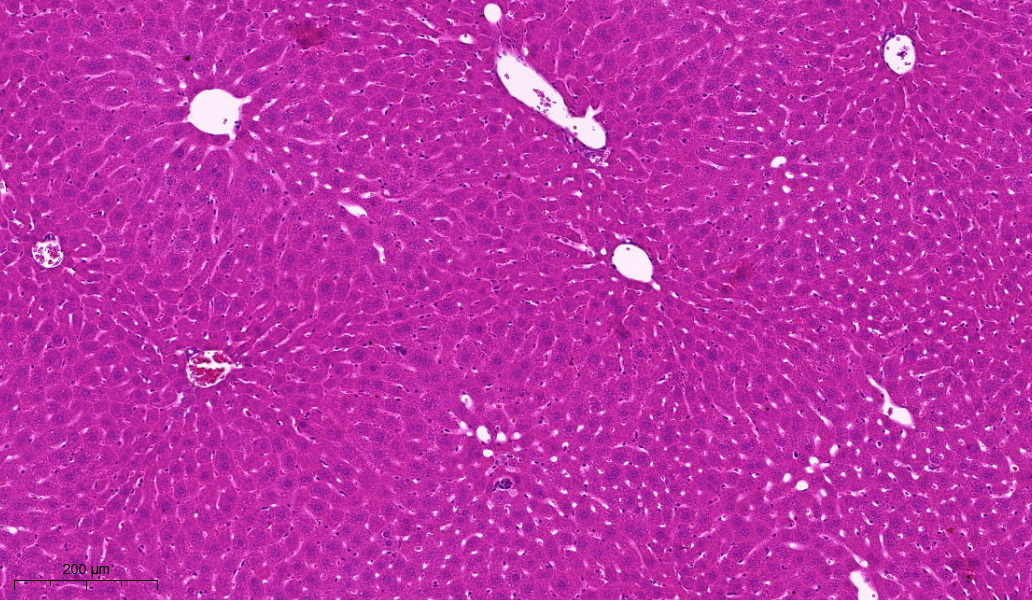

Supplement: Supplementary file 3 — Source Data Figs. 1–5 and Source Data Extended Data Figs. 1–10. [file 41586_2024_8165_MOESM3_ESM.zip › 2023-01-01106D-f1-5;sf1-10/Images/ExtendedData_Fig5/CCC_Liver.jpg]

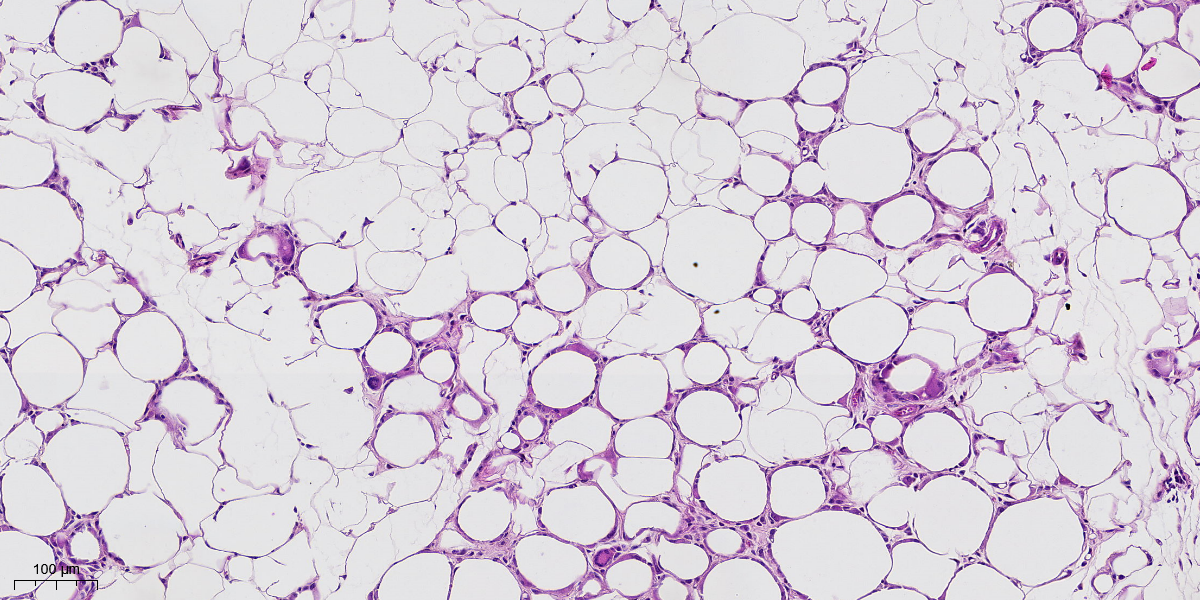

Supplement: Supplementary file 3 — Source Data Figs. 1–5 and Source Data Extended Data Figs. 1–10. [file 41586_2024_8165_MOESM3_ESM.zip › 2023-01-01106D-f1-5;sf1-10/Images/ExtendedData_Fig5/H_epiAT.tif]

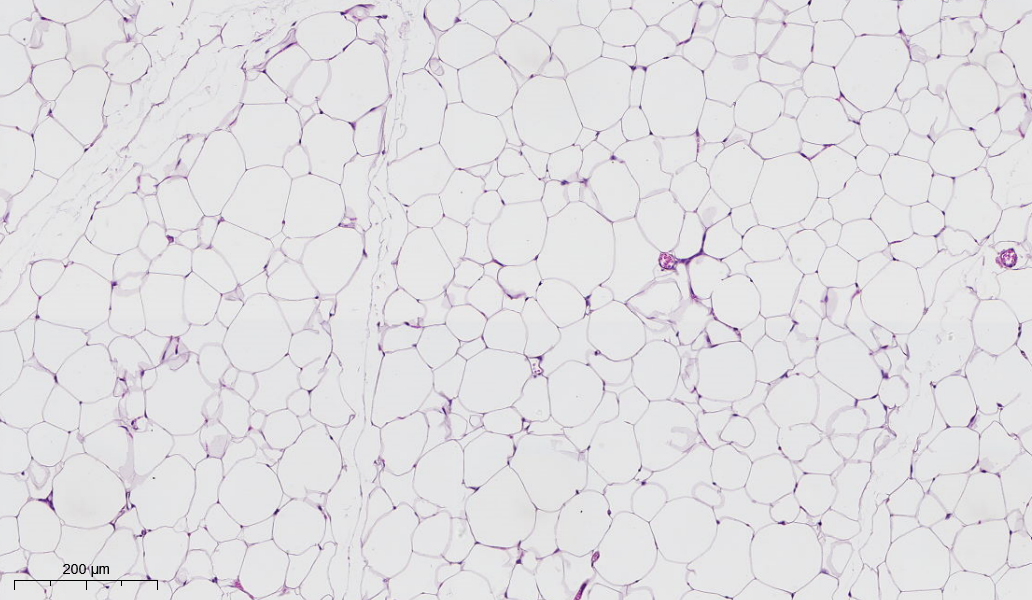

Supplement: Supplementary file 3 — Source Data Figs. 1–5 and Source Data Extended Data Figs. 1–10. [file 41586_2024_8165_MOESM3_ESM.zip › 2023-01-01106D-f1-5;sf1-10/Images/ExtendedData_Fig5/H_ingAT.tif]

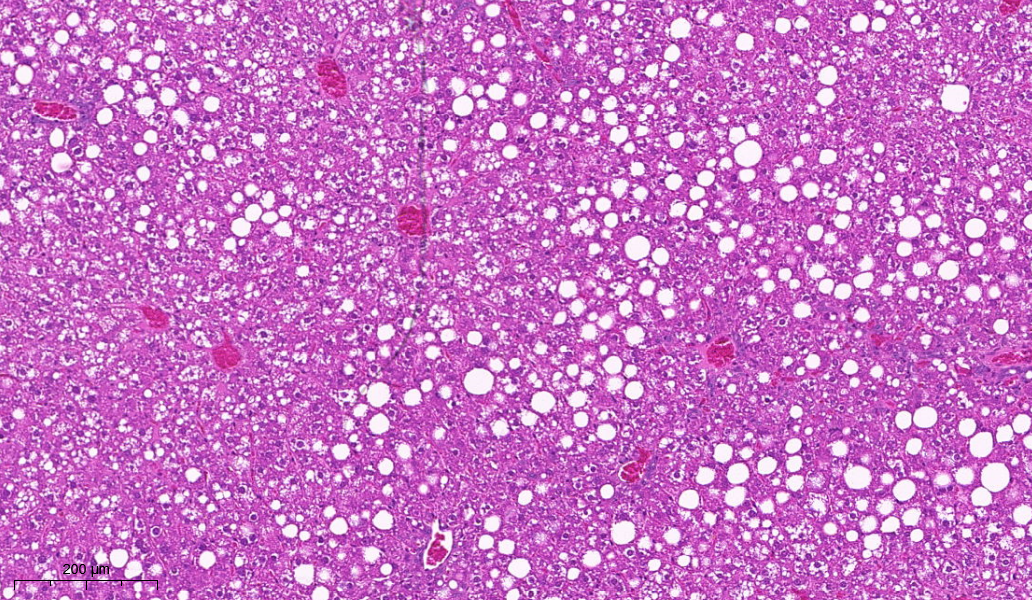

Supplement: Supplementary file 3 — Source Data Figs. 1–5 and Source Data Extended Data Figs. 1–10. [file 41586_2024_8165_MOESM3_ESM.zip › 2023-01-01106D-f1-5;sf1-10/Images/ExtendedData_Fig5/H_Liver.tif]

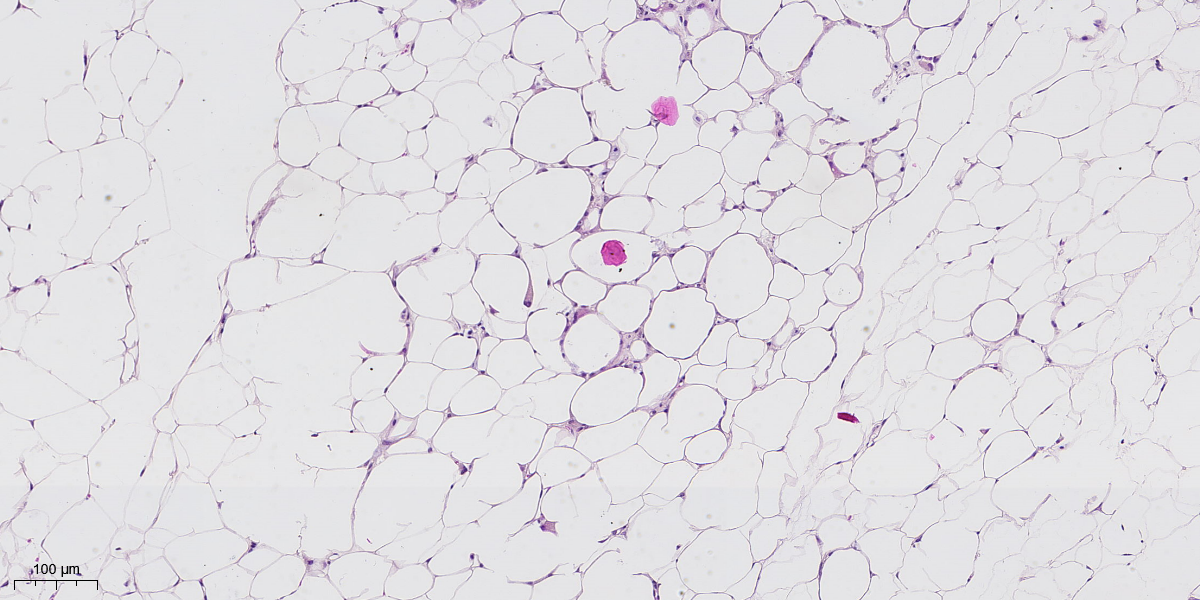

Supplement: Supplementary file 3 — Source Data Figs. 1–5 and Source Data Extended Data Figs. 1–10. [file 41586_2024_8165_MOESM3_ESM.zip › 2023-01-01106D-f1-5;sf1-10/Images/ExtendedData_Fig5/HC_epiAT.tif]

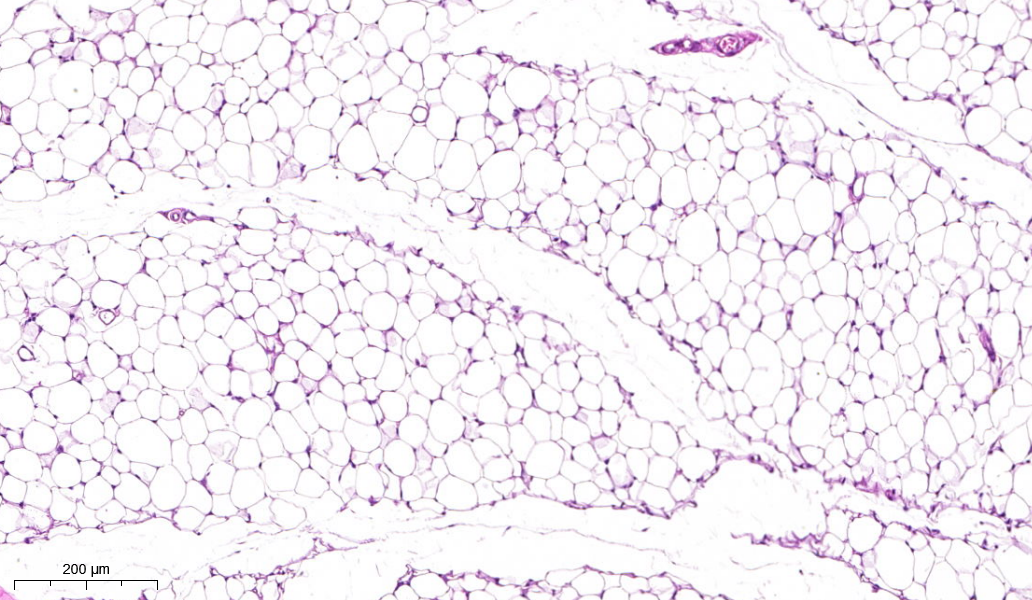

Supplement: Supplementary file 3 — Source Data Figs. 1–5 and Source Data Extended Data Figs. 1–10. [file 41586_2024_8165_MOESM3_ESM.zip › 2023-01-01106D-f1-5;sf1-10/Images/ExtendedData_Fig5/HC_ingAT.tif]

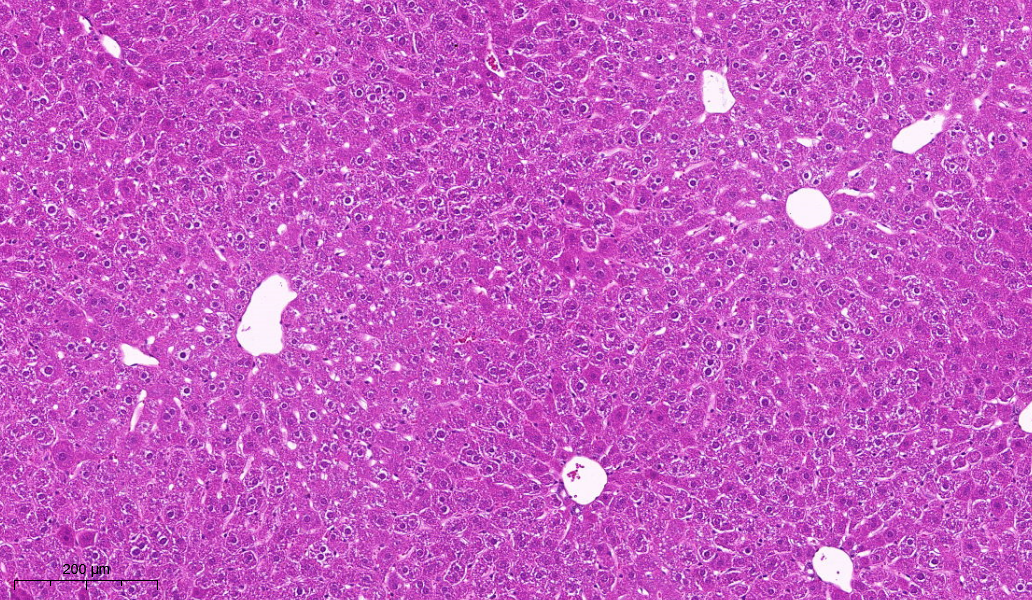

Supplement: Supplementary file 3 — Source Data Figs. 1–5 and Source Data Extended Data Figs. 1–10. [file 41586_2024_8165_MOESM3_ESM.zip › 2023-01-01106D-f1-5;sf1-10/Images/ExtendedData_Fig5/HC_Liver.tif]

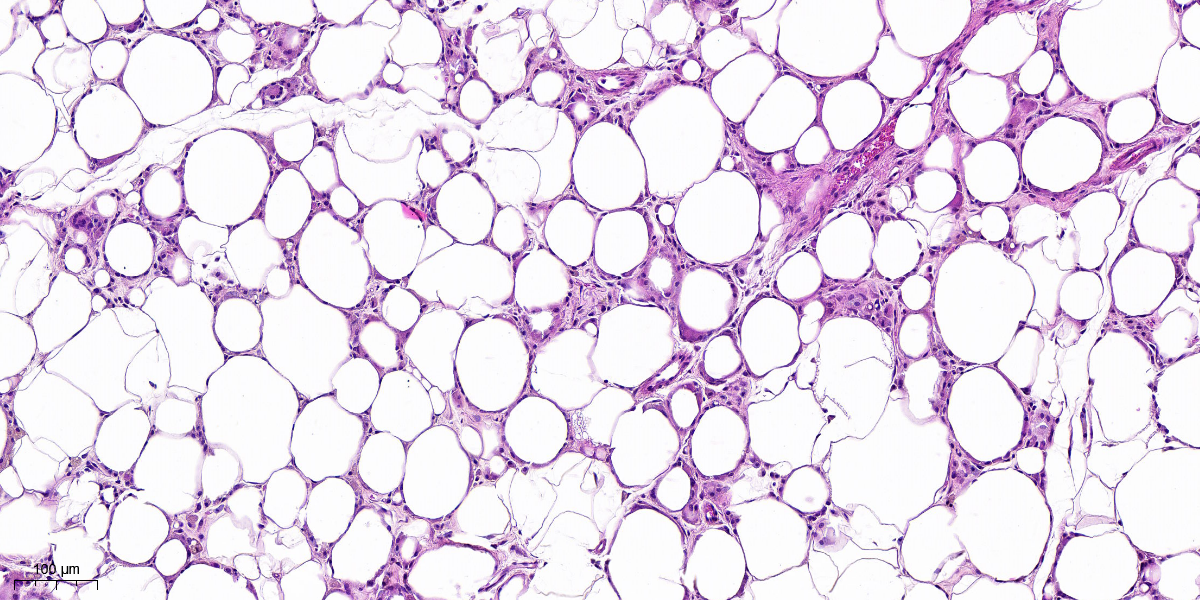

Supplement: Supplementary file 3 — Source Data Figs. 1–5 and Source Data Extended Data Figs. 1–10. [file 41586_2024_8165_MOESM3_ESM.zip › 2023-01-01106D-f1-5;sf1-10/Images/ExtendedData_Fig5/HH_epiAT.tif]

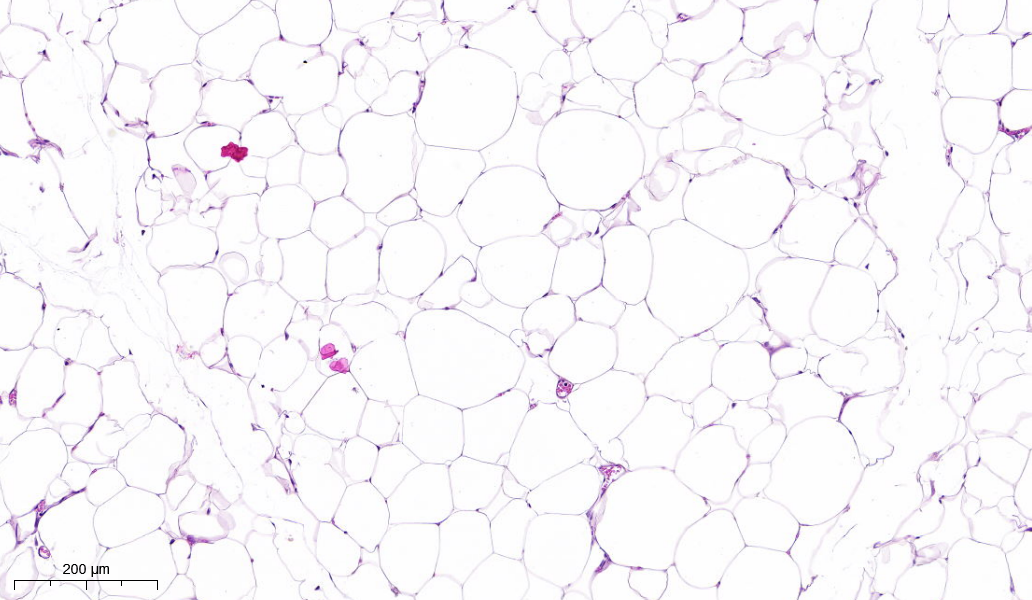

Supplement: Supplementary file 3 — Source Data Figs. 1–5 and Source Data Extended Data Figs. 1–10. [file 41586_2024_8165_MOESM3_ESM.zip › 2023-01-01106D-f1-5;sf1-10/Images/ExtendedData_Fig5/HH_ingAT.tif]

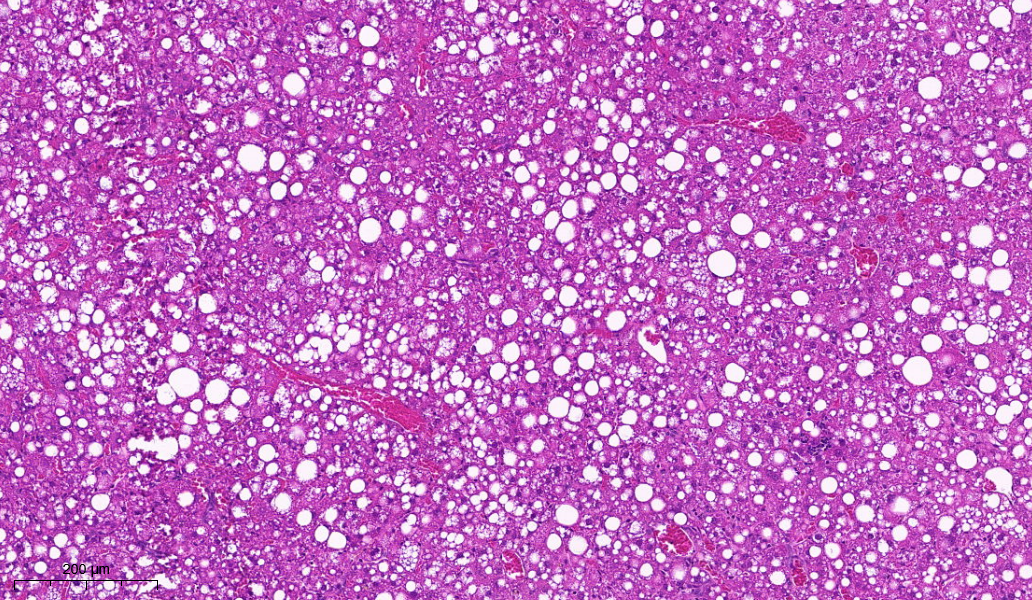

Supplement: Supplementary file 3 — Source Data Figs. 1–5 and Source Data Extended Data Figs. 1–10. [file 41586_2024_8165_MOESM3_ESM.zip › 2023-01-01106D-f1-5;sf1-10/Images/ExtendedData_Fig5/HH_Liver.tif]

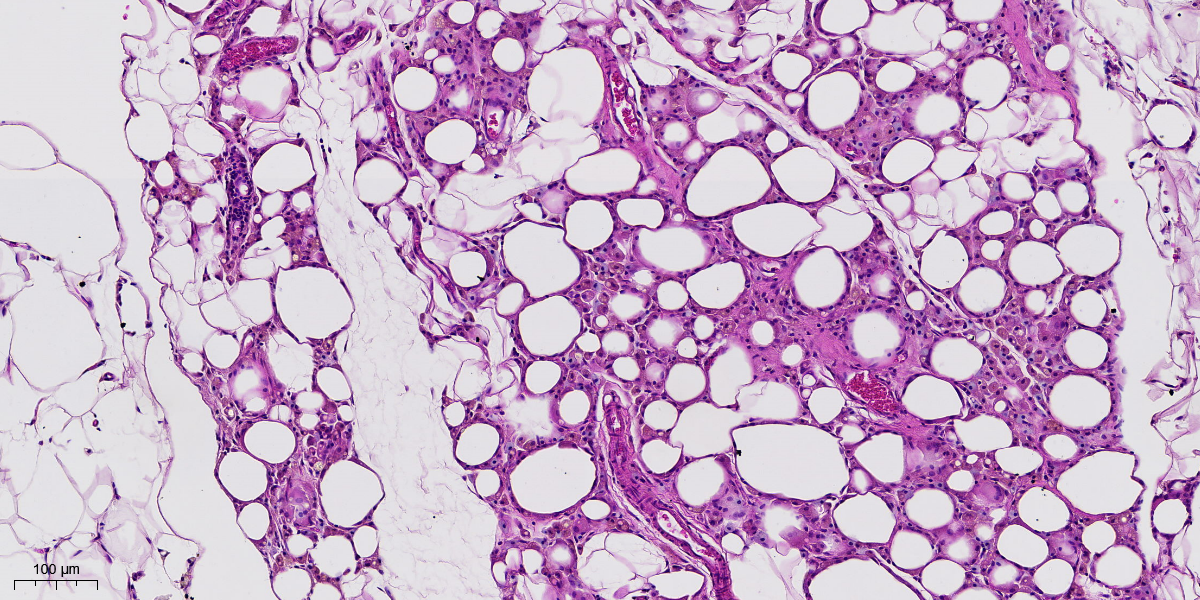

Supplement: Supplementary file 3 — Source Data Figs. 1–5 and Source Data Extended Data Figs. 1–10. [file 41586_2024_8165_MOESM3_ESM.zip › 2023-01-01106D-f1-5;sf1-10/Images/ExtendedData_Fig5/HHC_epiAT.tif]

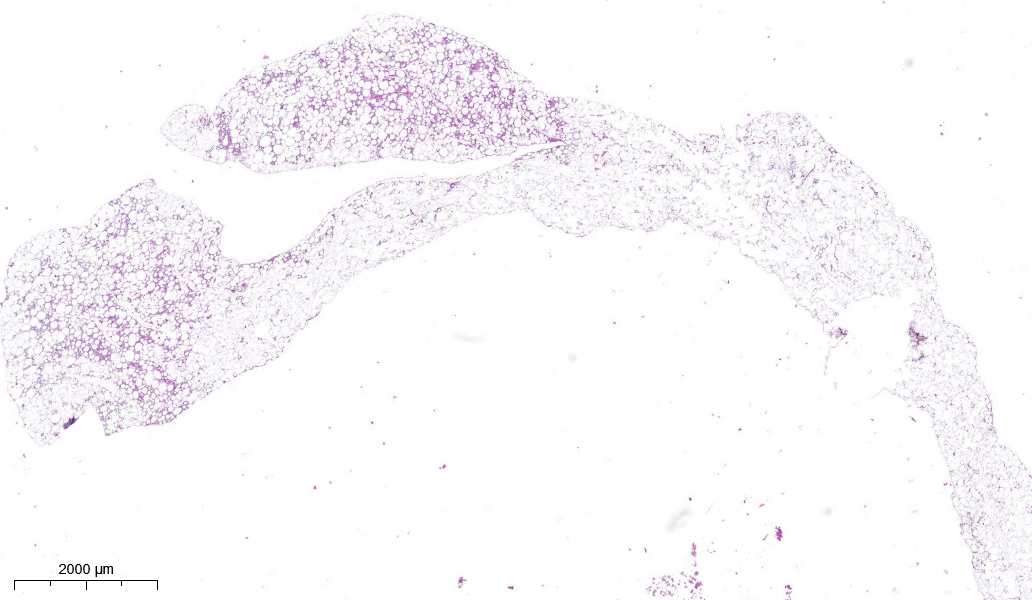

Supplement: Supplementary file 3 — Source Data Figs. 1–5 and Source Data Extended Data Figs. 1–10. [file 41586_2024_8165_MOESM3_ESM.zip › 2023-01-01106D-f1-5;sf1-10/Images/ExtendedData_Fig5/HHC_epiAT_large.tif]

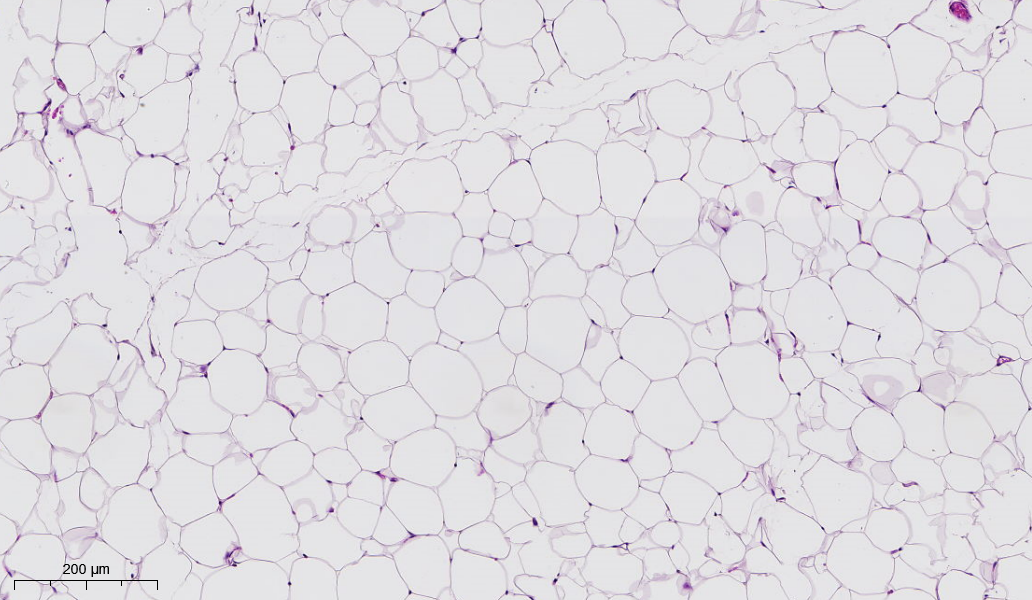

Supplement: Supplementary file 3 — Source Data Figs. 1–5 and Source Data Extended Data Figs. 1–10. [file 41586_2024_8165_MOESM3_ESM.zip › 2023-01-01106D-f1-5;sf1-10/Images/ExtendedData_Fig5/HHC_ingAT.tif]

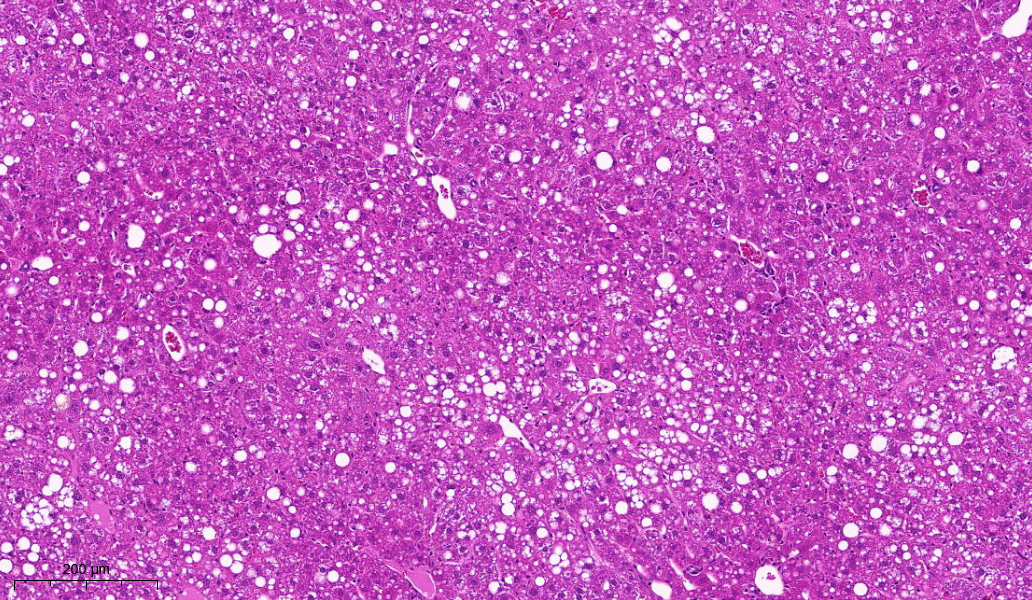

Supplement: Supplementary file 3 — Source Data Figs. 1–5 and Source Data Extended Data Figs. 1–10. [file 41586_2024_8165_MOESM3_ESM.zip › 2023-01-01106D-f1-5;sf1-10/Images/ExtendedData_Fig5/HHC_Liver.tif]

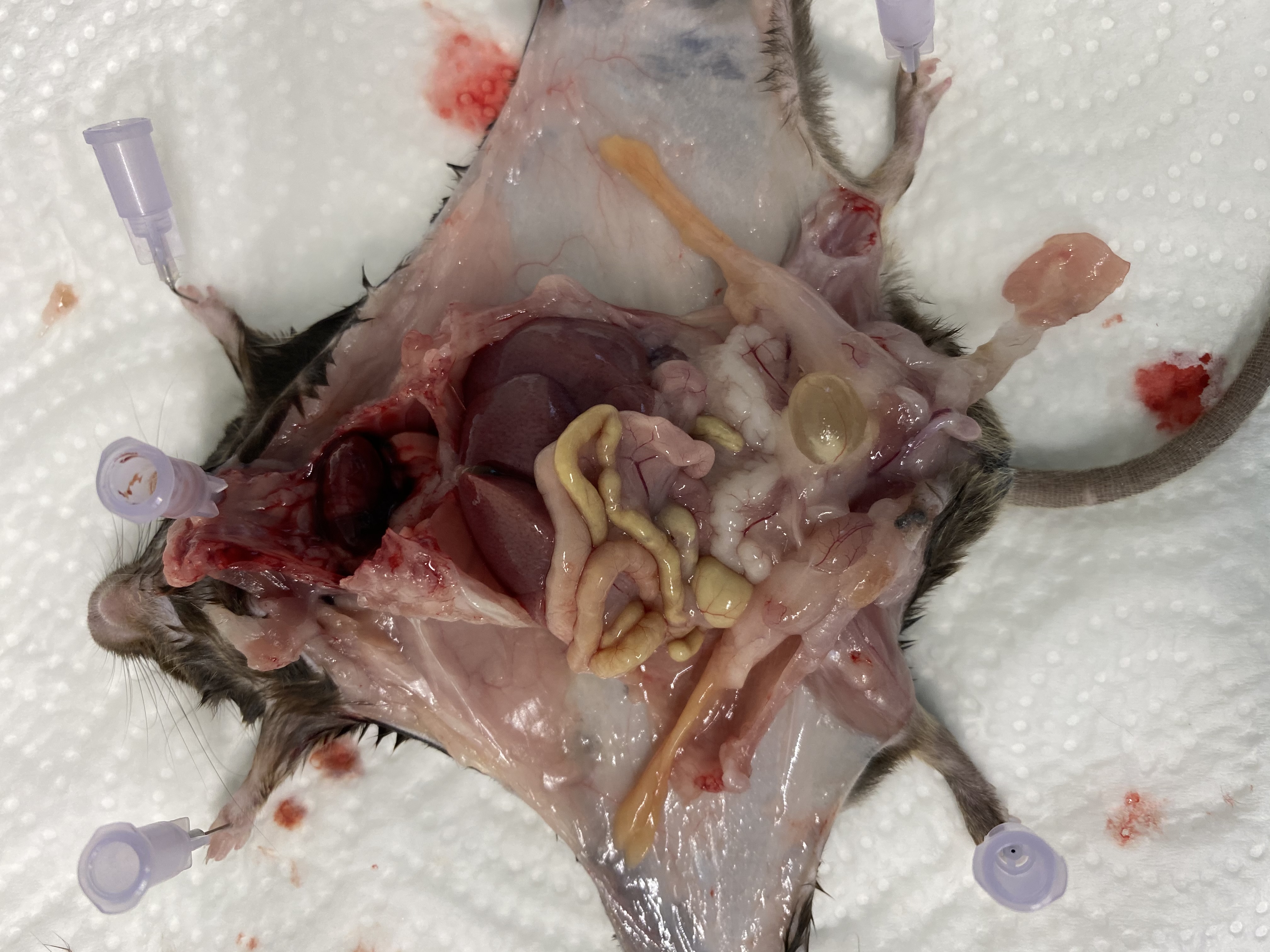

Supplement: Supplementary file 3 — Source Data Figs. 1–5 and Source Data Extended Data Figs. 1–10. [file 41586_2024_8165_MOESM3_ESM.zip › 2023-01-01106D-f1-5;sf1-10/Images/ExtendedData_Fig5/HHC_Mouse.jpg]
